# What did COVID‐19 pandemics teach us about single‐fraction radiotherapy for painful bone metastases—State of the art or undertreatment?

**DOI:** 10.1002/cam4.6231

**Published:** 2023-06-14

**Authors:** Tatjana Arsenijević, Aleksandar Stepanović, Brankica Milošević‐Maračić, Bojana Poparić‐Bandjur, Ivana Mišković, Dušica Gavrilović, Marina Nikitović

**Affiliations:** ^1^ University of Belgrade, Faculty of Medicine Belgrade Serbia; ^2^ Institute for Oncology and Radiology of Serbia Belgrade Serbia

## Abstract

**Background:**

Choosing the optimal treatment approach for patients with painful bone metastases during the COVID‐19 pandemic became challenging. A simple technique, single fraction radiotherapy was recommended for these patients usually referring to bone metastases as a single entity, although it is a very heterogeneous group of patients.

**Aim:**

This study aimed to analyze the response to palliative single fraction radiotherapy in relation to age, performance status, primary tumor, histopathology, and bone localization in the group of patients with painful bone metastases.

**Methods:**

A clinical, prospective, non‐randomized study was conducted at the Institute for Oncology and Radiology of Serbia, which included 64 patients with noncomplicated, painful bone metastases who underwent palliative, pain‐relieving radiation therapy with a single tumor dose of 8Gy in a single hospital visit. Response to treatment was patient reported via telephone interview using visual analog scale. The response assessment was based on the international consensus panel of radiation oncologists.

**Results:**

In the entire group of patients, 83% responded to radiotherapy. No statistically significant difference was observed in response to therapy, time to reach the maximum response, degree of pain reduction, nor in response duration depending on the patient's age, performance status, the primary origin of the tumor, histopathology, or location of the metastasis (bone) that was irradiated.

**Conclusion:**

Regardless of clinical parameters, palliative radiotherapy with a single dose of 8Gy can be considered very effective in quick pain relief in patients with noncomplicated painful bone metastases. Single fraction radiotherapy in a single hospital visit, as well as patient‐reported outcome for these patients may be considered favorable beyond Covid pandemics.

## 1 INTRODUCTION

Treatment options for patients with painful bone metastases nowadays are numerous, including radiotherapy, systemic therapy, surgery, and complete palliative and supportive care and pharmacotherapy. The complexity of choosing the optimal treatment approach for these patients in the context of efficiency, cost‐effectiveness, and quality of life is rising.

For decades, radiotherapy has been considered the most effective therapeutic method for painful bone metastases. Based on the results of numerous studies and meta‐analyses, the American Society of Therapeutic Radiology and Oncology (ASTRO) provided a guideline in 2017. with the conclusion that the radiotherapy technique of high radiation dose delivered in one fraction (so‐called “single shoot” technique) is equally effective in controlling pain in bone metastases as fractionated radiation regimens. However, duration of pain response, bone stability, and remineralization happen to be inferior after single fraction than after multifraction radiotherapy, leading to significantly higher re‐irradiation rate in the single‐fraction group of patients.^1^, ^2^, ^3^, ^4^, ^5^, ^6^, ^7^, ^8^, ^9^, ^10^, ^11^ Up to now, the optimal radiotherapy treatment regimen for achieving pain control still remains controversial among radiation oncologists, questioning everything from cancer‐related features to clinical studies design.^12^

With the COVID‐19 outbreak, oncology departments throughout the world were challenged, especially when it comes to patients needing palliative radiotherapy. At that point, some authors suggested that palliative radiotherapy should be limited only to emergencies,^13^ while others insisted that both palliative and curative patients should meet the same priority.^14^ As the initial impulse to switch bone metastases pain control to predominantly analgesic administration failed to demonstrate best efficiency due to multiple dose correction, escalations, and toxicity, palliative radiotherapy needed to be prioritized and optimized.^14^

Following guidelines during COVID‐19 pandemics, patients with painful bone metastases are recommended to undergo a simple technique, 8Gy single‐fraction radiotherapy in only one hospital visit, with telephone follow‐up whenever possible.^15^ Painful bone metastases were seen as a single entity, although it is a very heterogeneous group of patients, primary tumor sites, and histopathological diagnoses, as well as localization of metastases on the skeleton. The question imposed, whether that change in palliative radiotherapy was optimal, or some patients experienced undertreatment?^16^

Therefore, we set the goal to analyze the response to treatment concerning age, performance status (PS) of the patient, primary tumor, histopathology, and localization of metastases in the group of patients with painful bone metastases who underwent palliative, pain‐relieving, 8Gy “single shoot” radiotherapy. The analysis of these clinical parameters might give another perspective in selection of patients with painful bone metastases for an optimal palliative approach.

## 2 METHODS

In the period from October 2019 to June 2020, a clinical, prospective, non‐randomized study was conducted at the Institute for Oncology and Radiology of Serbia, which included 64 patients with painful bone metastases. All patients were treated based on the current protocols for the diagnosis and treatment of malignant diseases, and the treatment was proposed by a multidisciplinary tumor board. Also, all patients were informed about the treatment protocol and gave written informed consent for participation in the study.

### 2.1 Study population

The study enrolled patients older than 18 years, with histopathologically verified any malignant disease, with radiographically and/or histopathologically confirmed bone metastases (one or more), previously treated or not treated with specific oncological therapy, and whose expected survival time was longer than 1 month. Patients with complicated bone metastases (spinal cord compression and pathological fracture), patients previously irradiated for the same bone metastases, and those with an expected survival time of <1 month were excluded from the study.

### 2.2 Pain assessment

All patients were clinically examined with an assessment of their performance status according to the ECOG performance scale.^17^ Pain intensity was assessed based on the ordinal scale for pain (VAS‐visual analog scale),^18^ and the use of analgesics was assessed according to WHO recommendations.^19^

### 2.3 Radiotherapy

All patients underwent palliative, pain‐relieving radiation therapy using the “single shoot” technique with a tumor dose of 8Gy in one fraction, with high‐energy photons (6MeV, 8MeV) on a linear accelerator. Radiation fields were determined individually for each patient based on reference bone X‐ray, CT, or MR images, and according to ICRU 62 recommendations.^20^

Radiotherapy was delivered in a “single hospital visit” manner, that is, in the single hospital visit, patient was presented to the tumor board, delineated, planned for radiotherapy, and irradiated.

### 2.4 Response to treatment

Pain intensity, performance status, as well as correction of analgesic use were evaluated first after 48 h following radiation, then once a week during the first 4 weeks, and then once a month until the third month after the radiation, using the same scales as before radiation. These assessments were carried out through telephone interviews or regular check‐ups, based on the standards of the international consensus panel of radiation oncologists^21^:

1. Complete response: the complete absence of pain after radiotherapy without additional analgesics.
2. Partial response: pain reduction of ≥2 on the ordinal pain scale without increasing the analgesic dose.
3. Absence of response to radiation therapy: pain intensity remained the same or worsened within 4 weeks of radiation.
4. Duration of therapeutic response was recorded from the date of maximal response to the date of pain relapse or until study closure or death.
5. Pain relapse: worsening of pain by ≥2 on the ordinal pain scale after initially achieving response.
6. For each patient in our study, follow‐up lasted 3 months from the date of irradiation or until the date of death.

### 2.5 Statistical analyses

1. To examine the agreement of sample distributions with the normal distribution, graphs were used: normal QQ plot and histogram, as well as tests: Kolmogorov–Smirnov and Shapiro–Wilk.
2. To describe important parameters, and depending on their nature, measures of descriptive statistics were used: frequencies, percentages, mean value (average), median, standard deviation (SD), and range (range).
3. The value *α* = 0.05 was adopted for the level of statistical significance. In case of multiple testing on the same data set, Bonferroni correction of *α*‐value was used.
4. To test the differences between treatment groups, and depending on the nature of the examined parameters, the following were used: Fisher's exact test; Kruskal–Wallis test, Wilcoxon rank sum test.
5. The Kaplan–Meier product‐limit method was used to display the time to maximum response, time to progression, and overall survival, and survival analysis medians and corresponding 95% confidence intervals (95% CI) were used to describe them. The log‐rank test was used to test differences in time to maximum response and time to progression.
6. Standard tests in the statistical program R version 3.3.2 (2016‐10‐31) — “Sincere Pumpkin Patch”; Copyright (C) 2016 The R Foundation for Statistical Computing; Platform: x86_64‐w64‐mingw32/x64 (64‐bit) (Available at: www.r‐project.org; Retrieved: 2017‐01‐21) were used in data processing and analysis.

## 3 RESULTS

### 3.1 Patients characteristics

Table 1 shows the baseline characteristics of the patients included in our study.

**TABLE 1:** Patients characteristics at baseline (No 64).

Age (years)		
40–49	4	6.25%
50–59	15	23.44%
60–69	28	43.75%
70–79	13	20.31%
80–89	4	6.25%
Median (Range)	66 (43–87)	
Sex		
Female	26	40.63%
Male	38	59.37%
Performance status (ECOG PS)		
1	18	28.12%
2	19	29.69%
3	22	34.38%
4	5	7.81%
Primary tumor localization		
Breast	5	7.81%
Colon	5	7.81%
Lung	22	34.38%
Cervix	2	3.12%
Unknown primary	4	6.25%
Hypopharynx	2	3.12%
Prostate	6	9.37%
Kidney	6	9.37%
Uterus	1	1.56%
Penis	2	3.12%
Bladder	4	6.25%
Esophagogastric	1	1.56%
Liver	2	3.12%
Rectum	2	3.12%
Histopathology		
Adenocarcinoma	21	32.81%
Squamous cell	9	14.06%
Small cell	7	10.94%
Unknown	7	10.94%
Ductal	3	4.69%
Renal cell	5	7.81%
Endometrial	1	1.56%
Transitional cell	6	9.37%
LCNEC	1	1.56%
NET	2	3.12%
HCC	1	1.56%
Lobular carcinoma	1	1.56%
Pain intensity before RT (VAS)		
No pain (0–2)	0	0.00%
Mild pain (3–4)	19	29.69%
Moderate pain (5–7)	22	34.38%
Severe pain (8–10)	23	35.94%
Analgesics		
No analgesics	7	10.94%
NSAID	21	32.81%
Weak opioids	11	17.19%
Strong opioids	25	39.06%
Localization of the metastases		
Pelvis	16	25.00%
Spinal vertebrae	37	57.81%
Humerus	6	9.37%
Femur	4	6.25%
Ribs	1	1.56%

Abbreviations: HCC, hepatocellular carcinoma; LCNEC, large‐cell neuroendocrinic tumor; NET, neuroendocrinic tumor; NSAID, nonsteroid anti‐inflammatory drugs; RT, radiotherapy; VAS, visual analog scale.

Out of the 64 patients included in the study, 38 were men (59%) and 26 were women (41%), with an average age of 66 years (range 43–87 years). Even a third of the patients (34%) were ECOG performance status 3 (PS3). Based on the primary location of the tumor and histological type, the group was very heterogeneous with almost 35% of patients having confirmed primary lung cancer. The largest number of cases (33%) was adenocarcinoma. More than half of the patients in our group (58%) had metastases in the spinal column, and 25% of the patients had metastases in the pelvis. Very strong pain (VAS 8–10) had the largest number of patients, 23 of them (36%), of which 6 (10%) had a severity of 10 on the VAS. Almost 40% of patients used strong opioids to control the pain.

### 3.2 Response to radiotherapy

In the entire group of 64 patients, 53 of them (83%) responded to radiotherapy, while 11 patients (17%) did not.

Of the 53 radiation responders, 19 (36%) achieved a complete response, that is, the complete absence of pain. In the other 34 patients (64%), a maximum partial response was noted with a median difference in pain reduction of 4 points on the VAS (rank 3–6). The median time to maximal response was 25 days (range 2–93 days), or an average of 3 weeks (0–13 weeks).

### 3.3 Analysis of response to radiotherapy in relation to predictive factors

In our group of patients, no statistically significant difference was observed in the response to therapy depending on the patient's age (Fisher's exact test *p* = 0.39), performance status (Fisher's exact test *p* = 1), the primary origin of the tumor, that is, histological type of tumor (Fisher's exact test *p* = 0.17), nor depending on the location of the metastasis (bone) that was irradiated (Fisher's exact test *p* = 0.999) (Table 2).

**TABLE 2:** Response to radiotherapy concerning the patient's age, performance status, primary origin (histological type of tumor), and localization of the metastases that were irradiated.

Patients age	40–50 years	50–60 years	60–70 years	70–80 years	80–90 years		
No response to radiotherapy	1 (25.00%)	1 (6.67%)	7 (25.00%)	1 (7.69%)	1 (25.00%)		
With response to radiotherapy	3 (75.00%)	14 (93.33%)	21 (75.00%)	12 (92.31%)	3 (75.00%)		
Total	4 (100%)	15 (100%)	28 (100%)	13 (100%)	4 (100%)		Fisher Exact Test: *p* = 0.39 (*p* > 0.05)

Performance status	PS1	PS2	PS3	PS4			
No response to radiotherapy	3 (16.67%)	3 (15.79%)	4 (18.18%)	1 (20.00%)			
With response to radiotherapy	15 (83.33%)	16 (84.21%)	18 (81.82%)	4 (80.00%)			
Total	18 (100%)	19 (100%)	22 (100%)	5 (100%)			Fisher's exact test: *p* = 1 (*p* > 0.05)

Histological type (primary origin)	Breast	Gastro intestinal	Lungs	Gynecological	Unknown primary	Urological	
No response to radiotherapy	1 (20.00%)	4 (33.33%)	1 (4.55%)	1 (33.33%)	0 (0.00%)	4 (22.22%)	
With response to radiotherapy	4 (80.00%)	8 (66.67%)	21 (95.45%)	2 (66.67%)	4 (100%)	14 (77.78%)	
Total	5 (100%)	12 (100%)	22 (100%)	3 (100%)	4 (100%)	18 (100%)	Fisher's exact test: *p* = 0.17 (*p* > 0.05)

Localization of metastases irradiated	Spinal column	Pelvis	Femur + humerus				
No response to radiotherapy	3 (18.75%)	6 (16.22%)	2 (20.00%)				
With response to radiotherapy	13 (81.25%)	31 (83.78%)	8 (80.00%)				
Total	16 (100%)	37 (100%)	10 (100%)				Fisher's exact test: *p* = 0.999 (*p* > 0.05)

**FIGURE 1:**
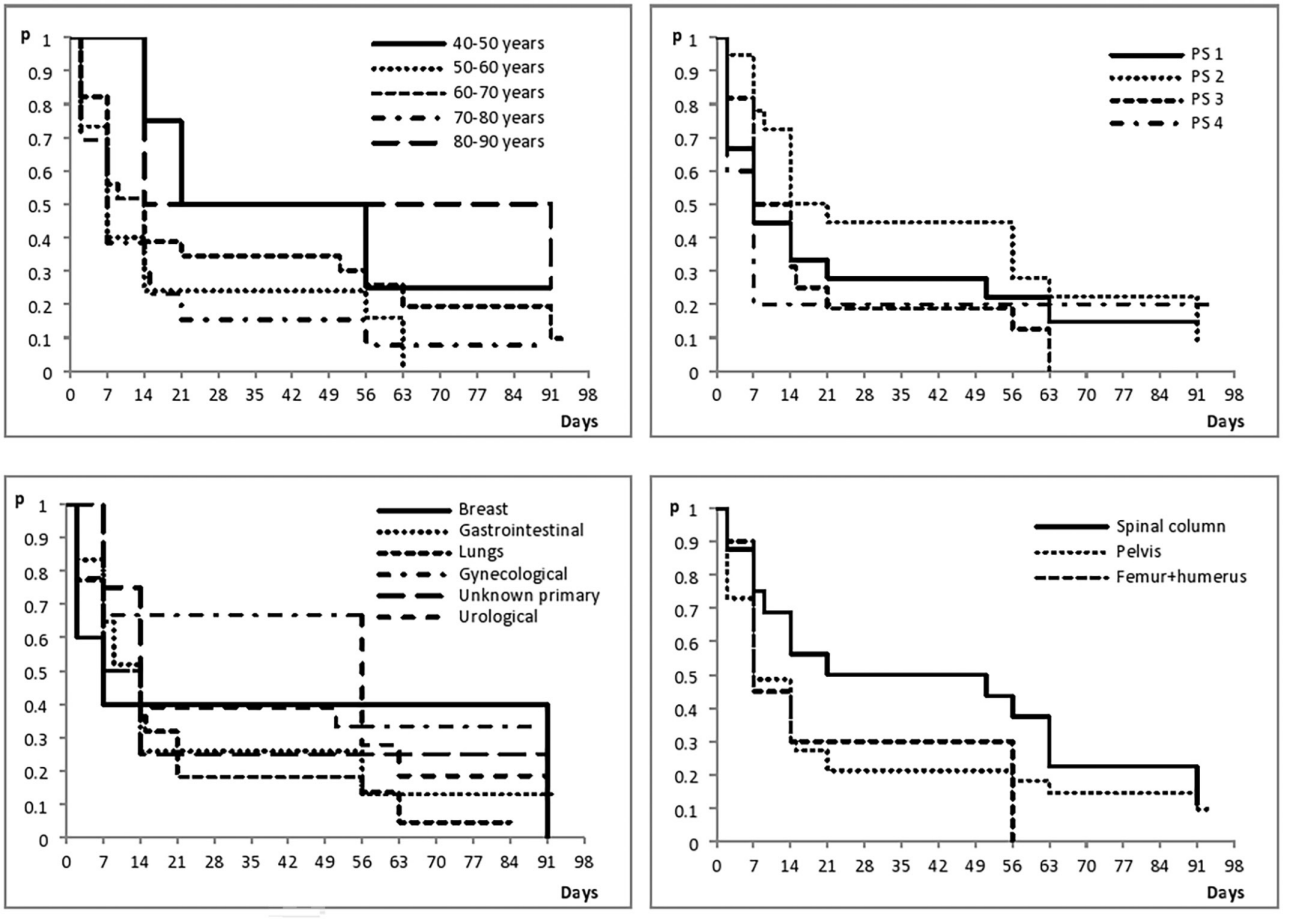
Time to response in relation to the patient's age, performance status, primary origin (histological type of tumor), and localization of the metastases that were irradiated.

Also, no statistically significant difference was observed in the time to reach the maximum response according to any parameter: age of the patient (log‐rank test, *p* = 0.138), performance status (log‐rank test, *p* = 0.488), the primary origin of the tumor, that is, histological type of tumor (log‐rank test, *p* = 0.77), as well as depending on the location of the metastasis (bone) that was irradiated (log‐rank test, *p* = 0.288). (Figure 1).

In the entire group of patients, there was no statistically significant difference in the level of response to radiotherapy (degree of pain reduction based on VAS) according to all the mentioned parameters: age of the patient (Kruskal–Wallis test = 4.92, *p* = 0.295), performance status (Kruskal–Wallis test = 0.17, *p* = 0.98), primary tumor origin (Kruskal–Wallis test = 7.31, *p* = 0.19), and metastasis localization (Kruskal–Wallis test = 0.54, *p* = 0.76). (Table 3).

**TABLE 3:** Level of response to radiotherapy in relation to the patient's age, performance status, primary origin (histological type of tumor), and localization of the metastases that were irradiated.

Patients age	40–50 years	50–60 years	60–70 years	70–80 years	80–90 years		
Number of patients	4	15	28	13	4		
Average	4.25	4.27	2.89	3.69	2.00		
SD	3.30	2.79	2.63	2.10	1.83		
SE	1.65	0.72	0.50	0.58	0.91		
Median	4.5	4	3	4	2		
Min‐Max	0–8	0–10	0–10	0–7	0–4		Kruskal–Wallis test = 4.92 *p* = 0.295

Performance status	PS1	PS2	PS3	PS4			
Number of patients	18	19	22	5			
Average	3.33	3.16	3.50	4.20			
SD	2.22	2.41	2.82	3.90			
SE	0.52	0.55	0.60	1.74			
Median	3	3	4	3			
Min–Max	0–9	0–8	0–10	0–10			Kruskal–Wallis test = 0.17 *p* = 0.98

Histological type (primary origin)	Breast	Gastrointestinal	Lungs	Gynecological	Unknown primary	Urological	
Number of patients	5	12	22	3	4	18	
Average	4.80	2.83	4.18	2.67	3.00	2.67	
SD	3.63	3.66	2.22	2.31	1.15	1.94	
SE	1.62	1.06	0.47	1.33	0.58	0.46	
Median	4	1	4	4	3	2.5	
Min‐Max	0–10	0–10	0–9	0–4	2–4	0–6	Kruskal–Wallis test = 7.31 *p* = 0.19

Localization of metastases irradiated	Spinal column	Pelvis	Femur + humerus				
Number of patients	16	37	10				
Average	3.88	3.35	2.90				
SD	3.18	2.41	2.47				
SE	0.80	0.40	0.78				
Median	4	3	2				
Min–Max	0–10	0–10	0–7				Kruskal–Wallis test = 0.54 *p* = 0.76

Abbreviations: Max, maximal reported pain according to visual analog scale; MIN, minimal reported pain according to visual analog scale; SD, standard deviation; SE, standard error.

For each patient in our study, follow‐up lasted 3 months from the date of irradiation or until the date of death. In that interval, out of 64 patients in our group, 19 of them had a progression of pain. The average time until the progression of pain in the entire group of patients was 34 days (1–69 days), that is, the duration of the response was an average of 1 month after reaching the maximum response.

**FIGURE 2:**
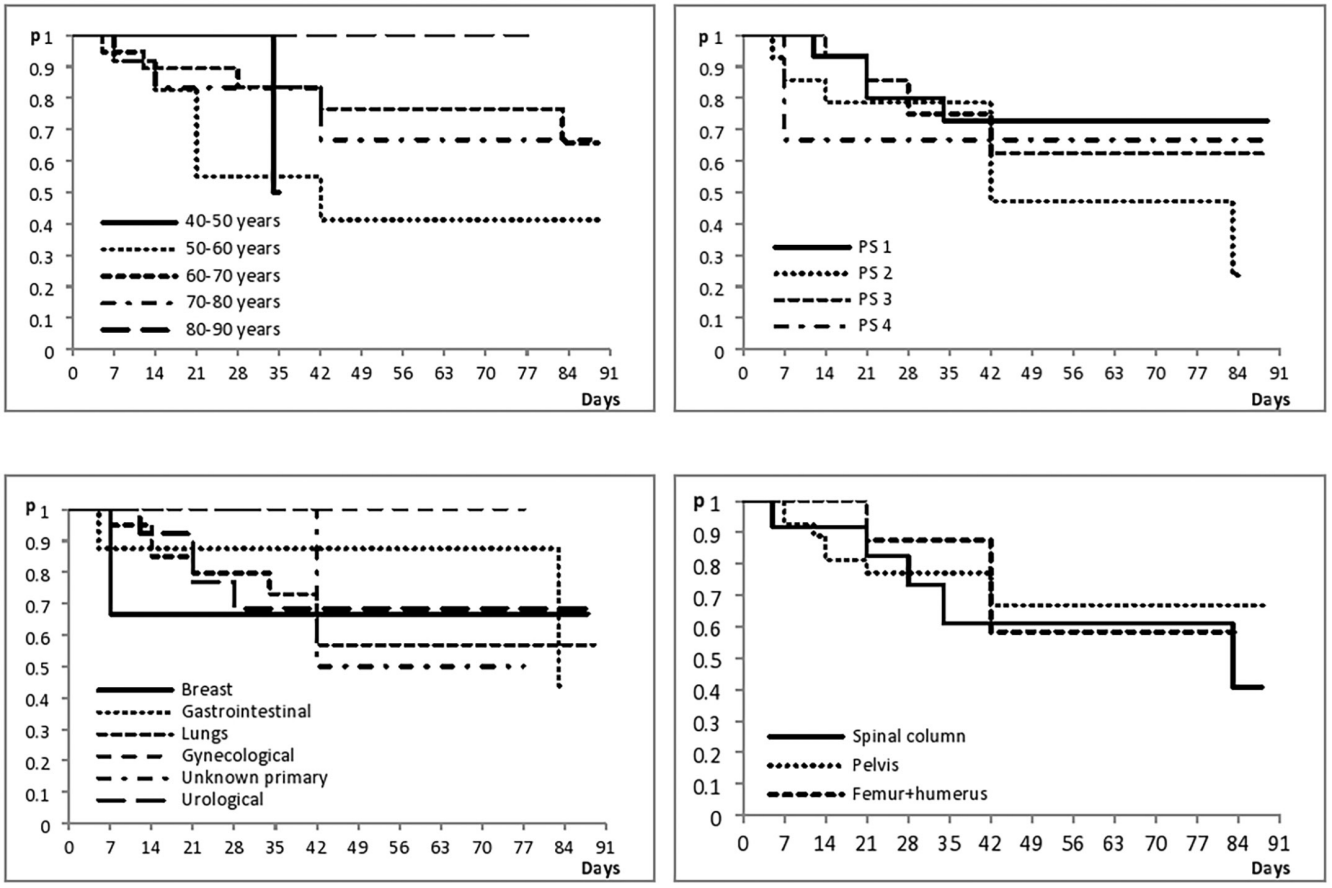
Pain progression in relation to the patient's age, performance status, histological type of tumor, and localization of the metastases that were irradiated.

In the entire group of patients, in the mean follow‐up time of 3 months, no statistically significant difference was observed in the time to pain progression after radiation depending on the patient's age (log‐rank test *p* = 0.39), performance status (log‐rank test *p* = 0.64), and the primary origin of the tumor, that is, histological type of tumor (log‐rank test *p* = 0.98), as well as depending on the location of the metastasis (bone) that was irradiated (log‐rank test *p* = 0.70). (Figure 2).

### 3.4 Overall survival

The average overall survival in the entire group of patients was 12 weeks (range 1–18 weeks). By the end of the study, 26 patients had died (41%), while almost two‐thirds (38 of them) were still alive (61%).

## 4 DISCUSSION

Based on multiple clinical trials, meta‐analysis and guidelines, “simple”, high dose, single fraction radiotherapy is considered an effective, short‐term regimen that enables quick and long‐lasting pain relief. It can significantly improve a patient's performance status and quality of life, with no delay in other oncological treatment and reduce the use of analgesics. It does, however result in higher re‐irradiation rate than with multi‐fractionation regimen and it is generally suitable for patients with uncomplicated bone metastases. With the use of modern techniques such as IMRT, Rapid Arc, SBRT, etc. the additional confusion was made in the treatment of patients with painful bone metastases regarding both optimal technique selection and the optimal tumor dose. Ultimately, with the COVID‐19 outbreak, radiation oncologists were challenged to quickly adjust the approach to these patients reintroducing fast regimen. But did we rush?

In the clinical practice as well as in most of the studies, palliative radiotherapy of painful bone metastases is usually seen as a single entity, although it is a very heterogeneous group of patients, the primary site of disease and histopathological diagnoses, as well as localization of metastases on the skeleton.^22^ In previous studies, the group of included patients was very heterogeneous (“any cancer”). Also, all studies that analyzed the antidolorous effect of radiotherapy focused on uncomplicated metastases (no spinal cord compression, no pathological fracture), in patients whose expected survival time was longer than 1 month.^23^, ^24^ Radiotherapy was performed on a linear accelerator with 6–10 MeV energy photons, except for superficial metastases on the rib when electron beams were used.^25^ Our study fully supports and maintains the consistency of the method with the mentioned studies, making it comparable with them.

In our study, the VAS scale was used to assess pain, and the response to radiotherapy was defined according to the recommendations of the radiotherapy consensus panel. Regardless of the applied pain assessment scale, in response to therapy, the definition of a complete response is the same everywhere—the complete absence of pain. The definition of a partial response, however, varies between studies but is defined as a reduction in pain by 1 category on a 4‐ or 5‐category scale, or by 2 points on the VAS. In this way, the studies are methodologically consistent, and our study fully corresponds to this system for assessing pain intensity. Also, in most studies, pain intensity was recorded 1, 2, 3, 4, 8, 12, and 24 weeks after irradiation, with which our study is in full agreement. Another important finding was that patient's compliance to telephone interview and patient reported outcome was 100% and we found it non‐challenging whatsoever. True, we did not have electronic patient‐reported outcome measures developed for that particular scenario.

The assessment of the patient's performance was based on the ECOG performance scale. In most previous studies, the inclusion criterion was Karnofsky performance scale (KPS) ˃40 (PS3), while in the study by Foro Arnalot et al., out of the 160 patients included, as many as 131 patients had KPS ≥70 (PS2).^26^ In our study more than a third of patients included were PS3, and five (8%) were completely immobile (PS4). Most of our patients had secondary deposits on the spinal vertebrae and pelvic bones, which is completely comparable with the studies of other authors.

In the entire group of 64 patients, 53 of them (83%) responded to radiotherapy. Of those 53 patients, 19 (36%) achieved a complete response, that is, complete absence of pain, of which 13 maintained a complete response without pain progression until the end of follow‐up. In 34 patients of our group (64%), a partial response to radiotherapy was recorded with a median difference in pain intensity of 4 points on the VAS (rank 3–6), which correlates with other series (average pain reduction—3.5 points on VAS).

In the meta‐analysis by Wu et al., which included eight clinical trials, the total response to a single shoot was 62%, while complete remission was recorded in 33% of patients.^27^ Comparable results were published by Sze et al. in which the total response was 60%, and the complete response was 34% in the single shoot group of patients.^28^ The results of the meta‐analysis by Chow et al. indicated a total response of 58% of patients, and a complete response in 23% of patients irradiated with TD 8Gy in one fraction.^29^ Our results are consistent with the results of other authors.

In the group of patients who responded to radiotherapy, the average time to reach the maximum response was 25 days (range 2–93 days), that is, an average of 3 eeks (0–13 weeks). The average time until pain progression was 34 days (1–69 days), that is, the response duration was an average of 1 month. In the Foro Arnalot study,^26^ the average time to maximal response was also 3 weeks, and the total duration of response was an average of 23 weeks.

Gaze et al. recorded a duration of response of 13.5 weeks, and Linden et al. of 18 weeks,^26^ which is significantly longer than our series (4 weeks). In the study by Nongkynrih et al., the average time to reach the maximum response was 4 weeks, but the duration of the maximum response was significantly longer than ours (22 months).^23^ However, we closed the study after 3 months of follow‐up. By that time 70% of our patients still had the treatment response.

In the available literature, there are very few comments regarding the patients who did not respond to radiation. In 11 patients of our study (17%), no reduction of pain was recorded, although six patients achieved stabilization of pain by the end of the study, which did not increase further. In these patients, there was no increase in the use of analgesics. These patients were women, predominantly elderly (median 72 years) and all had radioresistant malignancy (kidney, colon, and cervix). In five patients of this group, a rapid progression of pain after irradiation was noted.

Therefore, it is of great clinical importance to distinguish the group of patients who would benefit from radiotherapy compared to those who will not respond to it. Based on our results, no statistically significant difference was observed in the response to therapy depending on the age of the patient, performance status, primary origin of the tumor, histological type of the tumor, as well as depending on the location of the metastasis (bone) that was irradiated. There was no statistically significant difference in the time to pain progression, nor in the level of response to radiotherapy by any of the mentioned parameters. Even by separating the group of patients who had a response to radiotherapy (53 of them), no statistically significant difference was observed in the time to reach the maximum response according to any parameter. Several large‐world studies have analyzed the potential influence of pre‐therapeutic prognostic and predictive factors such as the patient's age, sex, and histology of the primary tumor and localization of metastases. None of these studies showed that any of these factors influenced response to therapy or survival.^30^

Overall survival in our entire group of patients was an average of 12 weeks (range 1–18 weeks), that is, 3 months. By the end of the study, 26 patients had died (41%), while almost two‐thirds (38 of them) were still alive (61%). In a retrospective study by Liu et al., 537 patients previously irradiated for painful bone metastases were examined. Median survival from irradiation was 6 months, which is longer than in our series. However, in this study, a significantly larger number of patients were in good performance status, in contrast to our study. Among other things, this study also concluded that patients with KPS below 80% have significantly worse survival. The worst survival was recorded in the gastrointestinal cohort, as well as in the lungs (4 months), which is correlated with our results, considering that in our study, patients with lung cancer were the most represented.^31^

The whole paradigm in the palliative approach was challenged during COVID‐19 pandemic, favoring quick, but non‐inferior radiotherapy regimens.^32^ A very interesting concept of care was published by Razvi et al, in 2019 as a rapid response radiotherapy program (RRRP), that is, the quick approach to consultation, planning, and radiation treatment delivered in one single day, particularly for palliative cancer patients.^33^ Since our study was conducted during the pandemic breakout, our results fully support these concepts.

## 5 CONCLUSION

Palliative radiotherapy using the technique of radiation in one session with a dose of 8 Gy can be considered very effective in quickly relieving the pain syndrome in patients with painful bone metastases. It has not been proven that any of the mentioned predictive factors have an influence on the response to therapy, the level of response (degree of pain reduction), the time to reach the maximum response, as well as the time to the progression of pain. Single‐fraction radiotherapy in a single‐hospital visit, as well as patient‐reported outcome for these patients may be considered favorable beyond COVID pandemics.

## AUTHOR CONTRIBUTIONS

**Tatjana Arsenijević:** Conceptualization (equal); data curation (lead); investigation (lead); methodology (equal); writing – original draft (lead). **Aleksandar Stepanović:** Data curation (equal); writing – review and editing (equal). **Brankica Milošević‐Maračić:** Data curation (equal). **Bojana Poparić‐Bandjur:** Data curation (equal). **Ivana Mišković:** Methodology (equal). **Dušica Gavrilović:** Software (lead). **Marina Nikitović:** Conceptualization (equal); methodology (equal); writing – review and editing (equal).

## FUNDING INFORMATION

This research received no external funding or any specific grant from funding agencies in the public, commercial, or not‐for‐profit sectors.

## CONFLICT OF INTEREST STATEMENT

All authors declare no conflict of interest.

## ETHICAL APPROVAL STATEMENT

The study was conducted according to the guidelines of the Declaration of Helsinki. Ethical review and approval were waived in this study, as in this study no experiments or novel treatments were conducted on humans (patients). The methodology represents a standard treatment modality that does not need ethical approval. The decision to treat the patients in the manner described in the study was made by the multidisciplinary tumor board of the Institute for oncology and radiology of Serbia.

The manuscript is a part of the academic sub‐specialist thesis of T.A. approved by the Faculty of Medicine, University of Belgrade, Serbia, 2019 (Protocol number: 04 BR: 18‐UON‐15).

## INFORMED CONSENT

Written informed consent was obtained for all subjects involved in the study. All subjects were fully aware that the data will be published. Written informed consent included that statement too. No personal identifiers are presented in this study.

## PERMISSION TO REPRODUCE MATERIAL FROM OTHER SOURCES

No other sources material was reproduced in this study.

## Data Availability

All data are contained within the article. Any additional information or explanation is available from the corresponding author.
